# Inference and prediction of malaria transmission dynamics using time series data

**DOI:** 10.1186/s40249-020-00696-1

**Published:** 2020-07-16

**Authors:** Benyun Shi, Shan Lin, Qi Tan, Jie Cao, Xiaohong Zhou, Shang Xia, Xiao-Nong Zhou, Jiming Liu

**Affiliations:** 1 School of Computer Science and Technology, Nanjing Tech University, Nanjing, 211800 Jiangsu China; 2 College of Information Engineering, Nanjing University of Finance & Economics, NanjingJiangsu, 210003 China; 3 Department of Computer Science, Hong Kong Baptist University, Kowloon, Hong Kong; 4 Department of Pathogen Biology, School of Public Health, Southern Medical University, Guangzhou, 510515 Guangdong China; 5 National Institute of Parasitic Diseases, Chinese Center for Diseases Control and Prevention, Shanghai, 200025 China; 6 Key Laboratory of Parasite and Vector Biology, National Health Commission of the People Republic of China, Shanghai, 200025 China; 7 Chinese Center for Tropical Disease Research, Shanghai, 200025 China; 8 Shanghai, 200025 China

## Abstract

**Background:**

Disease surveillance systems are essential for effective disease intervention and control by monitoring disease prevalence as time series. To evaluate the severity of an epidemic, statistical methods are widely used to forecast the trend, seasonality, and the possible number of infections of a disease. However, most statistical methods are limited in revealing the underlying dynamics of disease transmission, which may be affected by various impact factors, such as environmental, meteorological, and physiological factors. In this study, we focus on investigating malaria transmission dynamics based on time series data.

**Methods:**

A data-driven nonlinear stochastic model is proposed to infer and predict the dynamics of malaria transmission based on the time series of prevalence data. Specifically, the dynamics of malaria transmission is modeled based on the notion of vectorial capacity (VCAP) and entomological inoculation rate (EIR). A particle Markov chain Monte Carlo (PMCMC) method is employed to estimate the model parameters. Accordingly, a one-step-ahead prediction method is proposed to project the number of future malaria infections. Finally, two case studies are carried out on the inference and prediction of *Plasmodium vivax* transmission in Tengchong and Longling, Yunnan province, China.

**Results:**

The results show that the trained data-driven stochastic model can well fit the historical time series of *P. vivax* prevalence data in both counties from 2007 to 2010. Moreover, with well-trained model parameters, the proposed one-step-ahead prediction method can achieve better performances than that of the seasonal autoregressive integrated moving average model with respect to predicting the number of future malaria infections.

**Conclusions:**

By involving dynamically changing impact factors, the proposed data-driven model together with the PMCMC method can successfully (i) depict the dynamics of malaria transmission, and (ii) achieve accurate one-step-ahead prediction about malaria infections. Such a data-driven method has the potential to investigate malaria transmission dynamics in other malaria-endemic countries/regions.

## Background

Disease surveillance systems play essential roles in the control, elimination and eradication of infectious diseases, as they monitor, forecast and record the spatial and temporal distributions of disease prevalence [1]. Based on the time series of disease prevalence, various statistical methods have been proposed to predict the number of disease cases, such as autoregressive integrated moving average (ARIMA) method [2] and exponential smoothing method [3]. Such methods rely heavily on the statistical patterns of historical surveillance data, which are limited in understanding the underlying dynamics of disease transmission. However, in reality, the natural transmission of an infectious disease depends on the complex interactions among three types of interactive agents: the disease pathogens and/or parasites, the host, and the transmission agents [4, 5]. Moreover, the dynamics of disease transmission may also be affected by various risk factors ranging from microscopic to macroscopic scale, such as environmental [6], physiological [7], climatic [8, 9], socioeconomic [10, 11], and human behavioral factors [12–16]. Therefore, to combat infectious diseases, it would be necessary and helpful for public health authorities to model the dynamics of disease transmission by involving various impact factors and make policies from a perspective of systems thinking [17, 18].

As one of the most serious and deadly infectious diseases, malaria is a mosquito-borne infectious disease that is widespread in the tropical and subtropical regions around the equator. According to the World Health Organization, in 2018 there were 228 million cases of malaria worldwide resulting in an estimated 405 000 deaths. Approximately 93% of the cases occurred in Africa [19]. In China, the implementation of malaria control measures for over 30 years has significantly reduced the overall burden in the past century. However, early in the 21st century, malaria reemerged, representing once again a severe public health threat especially in the remote and poverty regions, such as those in Yunnan province, with very limited intervention and medical resources. In 2006 and 2007, a total of more than 0.11 million confirmed and more than 0.13 million suspected cases were reported [20]. In this paper, taking the *Plasmodium vivax* situations in two counties, Tengchong and Longling, in Yunnan province, China, as case studies, we focus on modeling the dynamics of *P. vivax* transmission based on time series of historical malaria prevalence data.

Starting from the Ross model, a variety of compartmental models with different level of complexity has been proposed to understand the dynamics of malaria transmission, which take into consideration various impact factors, such as population size [21], climate [22, 23], human movement [24–27], and socio-economic factors [11, 28]. (For more information, please refer to reference [29]). However, since all factors evolved in such models are treated endogenously, they are limited in modeling open systems that involve dynamically changing external factors (e.g., temperature and rainfall). For example, existing studies have revealed that daily temperature can influence not only the gonotrophic cycle of mosquitoes [30–32], but also the sporogonic cycle of parasites [33]. Moreover, rainfall or humidity can also significantly influence the population size of mosquitoes [34–37]. Accordingly, to build early warning systems and predict malaria transmission potential, the notions of vectorial capacity (VCAP) and entomological inoculation rate (EIR) have been used to capture the impact of dynamically changing temperature and rainfall on the dynamics of malaria transmission [38–40]. Conceptually, the VCAP incorporates all information about mosquito population (e.g., human biting rate, life expectancy), which is defined as the number of potentially infective contacts a person makes, through the mosquito population, per day [29]. By considering disease prevalence in the human population, the EIR captures the rate of infectious bites per person per day.

In addition to those meteorological factors, many other factors can also indirectly affect malaria transmission dynamics, making it difficult to predict the number of potential malaria infections. For example, human movement can introduce malaria cases from high-transmission areas into previously low-transmission or malaria-free areas [24, 25]. Furthermore, the imported cases may cause the recurrence of malaria when the environment is suitable for local transmission [26, 27]. In this case, to forecast the future disease infections of a location, it would be necessary for a prediction model to take into consideration both the dynamics of malaria transmission and the uncertainty of malaria cases imported from other locations. In this paper, we present a data-driven nonlinear stochastic model to characterize the impact of dynamically changing meteorological factors on malaria transmission dynamics, as well as the uncertainty about imported cases caused by human movement. Specifically, the proposed model consists of three components: (i) a weather-driven transmission model describing the malaria transmission dynamics affected by dynamically changing temperature and rainfall, (ii) a periodic function that reflects the seasonality of imported cases, and (iii) a stochastic error term (noise). In the proposed model, there are several unknown and unmeasurable parameters, such as the human recovery rate and the force of infection. Therefore, we employ a recently developed method known as PMCMC to infer unknown model parameters by fitting the time series of malaria prevalence data. Based on the estimated model parameters, we can further make a one-step-ahead prediction about the number of future malaria infections.

## Methods

In this section, we present a data-driven nonlinear stochastic model to infer and predict the dynamics of *P. vivax* transmission in Tengchong and Longling, Yunnan province, China.

### Model description

The data-driven nonlinear transmission model consists of three components: a weather-driven component, a periodic function, and an error term. The weather-driven component describes the dynamics of malaria transmission using the notion of VCAP and EIR. Let *g* denote the per-capita daily death rate of a mosquito (i.e., the force of mortality). Then, the average lifespan of a mosquito is 1/*g*. By assuming that the survivorship is constant over the mosquito lifespan, the survival time of a mosquito follows an exponential distribution based on the hazard model. Accordingly, the probability of a mosquito survive through one whole day is *p*=*e*^−*g*^. Further, let *n* denote the sporogonic cycle length of the *Plasmodium*. Then, the probability of an infected mosquito be infectious is *e*^−*gn*^. Based on the Macdonald model [41], vectorial capacity at time *t* can be formulated as

1 $$ V_{t} = \frac{ma^{2}e^{-gn}}{g}=\frac{-ma^{2}p^{n}}{\ln{p}},   $$

where *m* is the average mosquito density per person, and *a* is the expected number of bites on humans per mosquito, per day (i.e., human feeding rate). In doing so, VCAP describes the expected number of infectious bites from all the mosquitoes after feeding on an infectious host, assuming that all the mosquitoes get infected when they bite the infectious host. Notably, the value of *V*_*t*_ can be estimated by dynamically changing temperature and rainfall, so it varies with time *t*.

By definition, VCAP only characterizes the environmental- and biological-driven malaria transmission risk or the receptivity of an area to malaria. It does not take into account parasite availability in the human population [40, 42]. To assess the risk of infection for humans, we further use the notion of EIR as a measure of the average number of infectious bites per person per day [29]. Let *x*_*t*_ denote the proportion of humans who are infectious at time *t* and *c* denote the transmission efficiency from infectious human to mosquito. Consequently, mosquitoes become infected at a rate of *acx*_*t*_. Then, the proportion of infected mosquitoes is a ratio of two waiting times $\frac {acx_{t}}{g+{acx}_{t}}$: the waiting time to either death or infection 1/(*g*+*acx*_*t*_), and the waiting time to infection among surviving mosquitoes 1/*acx*_*t*_ [7]. Accordingly, the proportion of infectious mosquitoes at *t* is given by the product of the probabilities of becoming infected and, surviving the incubation period *e*^−*gn*^, that is,

2 $$ z_{t} = \frac{acx_{t-1}}{g+{acx}_{t-1}}e^{-gn}.  $$

At time *t*, EIR can be formulated as

3 $$ \mathrm{EIR_{t}} = {maz}_{t} =\frac{ma^{2}{cx}_{t-1}e^{-gn}}{g+{acx}_{t-1}}.  $$

Based on Eq. 1, we have

4 $$ \mathrm{EIR_{t}} = {cV}_{t} x_{t-1} \frac{1}{1+{acx}_{t-1}/g}.  $$

If *x*_*t*_ is very small, we have EI*R*_*t*_≈*cV*_*t*_*x*_*t*−1_ [7].

Based on the above-mentioned formulation, we can then model the dynamics of malaria transmission. Let *r* denote human recovery rate from malaria and *V*_*t*_ denote the value of VCAP at *t*. The change in the proportion of human infections at *t* can be formulated as

5 $$ \begin{aligned} \triangle x_{t} & = b\mathrm{EIR_{t}}(1-x_{t-1})-{rx}_{t-1}\\ & \approx bc{x_{t-1}}V_{t}(1-x_{t-1})-{rx}_{t-1}.\\ \end{aligned}  $$

Here, *b* is the transmission efficiency from infectious mosquito to human after an infective contact. In this paper, we denote *β*=*bc* as the mutual transmission efficiency between human and mosquito. Accordingly, we have

6 $$ x_{t} = x_{t-1} + \triangle x_{t} = -\beta V_{t }x_{t-1}^{2}+(1-r+\beta V_{t})x_{t-1}.  $$

Here, the parameters *β* and *r* will be inferred from time series of malaria prevalence data.

In addition to malaria transmission dynamics within an area, the number of malaria infections can also be affected by many other factors. Especially in the border areas between China and Myanmar, imported cases are one of the most important factors. Existing studies have shown that the imported malaria cases in Tengchong have a seasonal pattern [9]. Therefore, in this paper, we formulate the data-driven stochastic model as a combination of the weather-driven component *α*_1_*x*_*t*_, a periodic function *α*_2_| sin(*ωπ*∗*t*)|, and an error term *ε*_*t*_. In summary, the data-driven nonlinear stochastic model is formulated as

7 $$ y_{t} = \alpha_{1} x_{t} + \alpha_{2} |\sin(\omega\pi*t)| + \epsilon_{t},  $$

where $\epsilon _{t} \sim \mathcal {N}(0,\sigma)$. Here, the parameters *α*_1_, *α*_2_, *ω*, and *σ* will be inferred from the time series of malaria prevalence data.

### Estimation of vectorial capacity

Existing studies have shown that temperature and rainfall can be used to assess the value of VCAP by estimating *a*, *p*, *n*, and *m* in Eq. 1. Denote *u* as the gonotrophic cycle length of mosquitoes, that is, the period between the blood meal and oviposition. Then, *u* can be estimated by the dynamically changing temperature *T* [40]:

8 $$ u={0.5+f_{u}/(T-g_{u}+2)},  $$

where *f*_*u*_ is the number of degree days needed for mosquito maturation, and *g*_*u*_ is the threshold below which gonotrophic development ceases. Accordingly, the daily human feeding rate can be calculated as *a*=*h*/*u*, where *h* is the proportion of mosquitoes that have ever fed on a human (i.e., human blood index). The probability of a mosquito surviving through one whole day can be formulated as *p*=*γ*^1/*u*^, where *γ* is the survival rate per cycle.

Ceccato et al. [40] have also shown that the length of gonotrophic cycle of mosquitoes *n* can be estimated by temperature *T* as

9 $$ n=\frac{f_{n}}{2f_{u}/u(T-g_{u}+2)+(T-g_{n})},  $$

where *f*_*n*_ is the number of degree days required for parasite development, and *g*_*n*_ is the threshold below which parasite development ceases. The descriptions and values of all parameters and variables are summarized in Table 1. It is worth noting that even though the average mosquito density per person is simply estimated by *m*=10*R* as in reference [40], it does not affect the results of our model. The reason is that *m* is multiplied by *β* and *α*_1_ in the proposed stochastic model, which will be inferred using time series of malaria prevalence data.

**Table 1:** Descriptions and values of model parameters and variables

Parameter	Description	Values
*y*_*t*_	Output of the data-driven stochastic model at time step *t*	CISDCP
*x*_*t*_	Output of the weather-driven model at time step *t*	Hidden
*T*	Average temperature	MODIS
*R*	Average rainfall	TRMM
*f*_*u*_	Number of degree days needed for mosquito maturation	36.5 [40]
*g*_*u*_	Threshold below which gonotrophic development ceases	9.9 [40]
*γ*	Probability of a mosquitoes surviving through a gonotrophic cycle	0.5 [40]
*f*_*n*_	Number of degree days required for parasite development	105 [33]
*g*_*n*_	Threshold below which parasite development ceases	18°C [40]
*h*	Human blood index	0.7 [40]
*g*	Per-capita daily death rate of a mosquito	
*u*	Gonotrophic cycle length	Equation 8
*a*	Human feeding rate	*a*=*h*/*u*
*p*	Probability of a mosquito survive through one whole day	*p*=*e*^−*g*^=*γ*^1/*u*^
*n*	Sporogonic cycle length	Equation 9
*m*	Mosquito density per person	*m*=10*R* [40]
*b*	Transmission efficiency from mosquito to human	
*c*	Transmission efficiency from human to mosquito	
*β*	Mutual transmission efficiency: *β*=*bc*	To be estimated
*r*	Human recovery rate	To be estimated
*α*_1_	Coefficient of the weather-driven model	To be estimated
*α*_2_	Magnitude of the seasonal effect	To be estimated
*ω*	Seasonality parameter	To be estimated
*σ*	Variance of observation noise	To be estimated

^1^CISDCP: China Information System for Disease Control and Prevention (http://www.phsciencedata.cn/Share/)

^2^MODIS: Moderate Resolution Imaging Spectroradiometer (https://modis.gsfc.nasa.gov/)

^3^TRMM: Tropical Rainfall Measuring Mission (https://gpm.nasa.gov/trmm)

### Inference of model parameters

In this section, we employ a particle Markow chain Monte Carlo (PMCMC) algorithm to infer the model parameters based on time series of malaria prevalence data **y**_1:*τ*_. Let *θ*={*β*,*r*,*α*_1_,*α*_2_,*ω*,*σ*} denote the set of parameters in the proposed model (see Table 1). We assume that the output *y*_*t*_ of the model follows a Gaussian distribution. Then, we can infer the values of model parameters in *θ* and the latent state variables **x**_1:*τ*_ using the Bayesian approach by calculating their posterior distribution

10 $$ p(\theta,\mathbf{x}_{1:\tau}|\mathbf{y}_{1:\tau})=\frac{p(\mathbf{y}_{1:\tau},\mathbf{x}_{1:\tau} |\theta)p(\theta)}{p(\mathbf{y}_{1:\tau})},  $$

where *p*(*θ*) is the prior distribution of parameters in *θ*.

Since the time series of malaria prevalence is discrete, the posterior distribution in Eq. 10 is analytically intractable. To solve this problem, the Markov chain Mento Carlo (MCMC) algorithm provides a way to avoid deriving the analytical solution of the posterior distribution by generating samples based on the prior and likelihood. Based on the MCMC algorithm, we need to evaluate the posterior distribution of *θ*^∗^ and **x**_1:*τ*_ given **y**_1:*τ*_ at the same time by computing the likelihood *p*(**y**_1:*τ*_,**x**_1:*τ*_|*θ*) and the prior *p*(*θ*). However, it is extremely challenging to choose an efficient proposal distribution for a nonlinear and high-dimensional model [43]. Therefore, in this paper, we adopt a particle MCMC algorithm to tackle this challenge, which is a combination of the MCMC and Sequential Monte Carlo (SMC) algorithms.

With respect to the PMCMC algorithm, the parameters in *θ*^∗^ are first sampled from a proposal density *q*(*θ*^∗^|*θ*) and then $\textbf {x}^{*}_{1:\tau }$ is indenpendently sampled from *p*(**x**_1:*τ*_|**y**_1:*τ*_,*θ*^∗^). The new values of $\textbf {x}_{1:\tau }^{*}$ and *θ*^∗^ will be accepted with the rate [44, 45]:

11 $$ AR = \min \left(\frac{\hat{p}(\mathbf{y}_{1:\tau}|\theta^{*})p(\theta^{*})}{\hat{p}(\mathbf{y}_{1:\tau}|\theta) p(\theta)}\frac{q(\theta|\theta^{*})}{q(\theta^{*}|\theta)}), 1\right),  $$

where the marginal likelihood $\hat {p}(\mathbf {y}_{1:\tau }|\theta ^{*})$ is estimated through the SMC algorithm. Moreover, the SMC algorithm can also provide an approximation for *p*(**x**_1:*τ*_|**y**_1:*τ*_,*θ*) by propagating particles based on the weather-driven model. The detailed MCMC procedure is shown in Algorithm 1. 
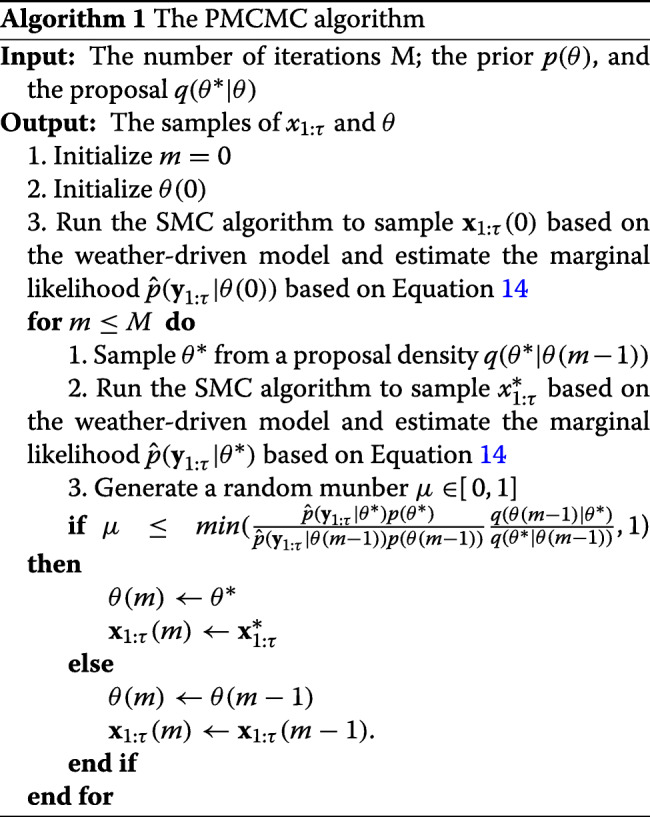


As one kind of particle filtering algorithm, the SMC algorithm allows us to numerically approximate the distribution of *p*(**x**_1:*τ*_|**y**_1:*τ*_,*θ*) by simulating the unknown trajectories of the variable *x*_*t*_ from the weather-driven model [45]. Given a number of *J* particles, the key idea behind the particle filtering is to update each particle sequence through time so that the weighted particles provide an approximation for *p*(**x**_1:*t*_|**y**_1:*t*_,*θ*) at any time *t*. First, the state of each particle $x^{j}_{t}$ at time *t* is simulated directly from the weather-driven model (Eq. 6). Then, each particle is filtered according to the observation model and assigned a weight $w_{t}^{j}$:

12 $$ w_{t}^{j}=p\left(y_{t}|x_{t}^{j},\theta\right) \sim \mathcal{N}(\alpha_{1}x_{t}+\alpha_{2}|\sin(\omega \pi *t)|,\sigma),  $$

which is simply the probability of observing *y*_*t*_ given the state of the particle $x_{t}^{j}$ and the estimated parameters (Eq. 7). By averaging the weight of all particles, we can approximate the conditional marginal likelihood *p*(*y*_*t*_|*y*_*t*−1_,*θ*) as

13 $$ p(y_{t}|y_{t-1},\theta)=\frac{1}{J}\sum_{j=1}^{J}w_{t}^{j}.  $$

Accordingly, the likelihood of observing the entire time series given *θ* can be approximated as

14 $$  \hat{p}(\mathbf{y}_{1:\tau}|\theta)=\prod_{t=1}^{\tau}p(y_{t}|y_{t-1},\theta).  $$

Such an approximation is further used to evaluate the acceptance probability (Eq. 11) in the MCMC procedure.

### One-step-ahead prediction

Once the model parameters *θ* are inferred based on time series of malaria prevalence, we can make a one-step-ahead prediction about future malaria infections. Suppose we have time series of malaria infections **y**_1:*t*_ at time *t*. The objective of the one-step-ahead prediction is to estimate the number of malaria infections $\hat {y}_{t+1}$ at *t*+1. To achieve this, we first calculate and sample the hidden state $\hat {x}_{t}$ based on the stochastic model. Then, the hidden state $\hat {x}_{t+1}$ at the next time *t*+1 can be estimated based on Eq. 6 using the set of estimated model parameters $\hat {\theta }$. In this case, the value of *y*_*t*+1_ can be predicted as

15 $$ \hat{y}_{t+1} = \hat{\alpha_{1}} \hat{x}_{t+1} + \hat{\alpha_{2}} |\sin(\hat{\omega}\pi*(t+1))|,  $$

where $\hat {\alpha _{1}}$, $\hat {\alpha _{2}}$, and $\hat {\omega }$ are estimated as the mean values of the sampled parameters based on the PMCMC algorithm.

### Data collection and experimental settings

In this paper, we carry out case studies on the inference and prediction of malaria transmission dynamics in two counties, Tengchong and Longling, in Yunnan province, China. We use daily *P. vivax* cases in Tengchong and Longling from January 1, 2007, to December 31, 2010, which can be obtained from the China Information System for Disease Control and Prevention by application (CISDCP: http://www.phsciencedata.cn/Share/). Taking into consideration the incubation period of *P. vivax*, we aggregate the daily *P. vivax* cases every 16 days during the case study. In this case, there are 23 time periods every year. To train the proposed stochastic model, we use the prevalence data from January 1, 2007, to December 31, 2009. Then, we evaluate the performance of our model by predicting the number of *P. vivax* infections in 2010. Because there are only very few cases in the first three months in 2010, we start our prediction from April 2010.

The value of VCAP is calculated based on the dynamically changing temperature and rainfall in the proposed model. By carefully selecting representative areas of Tengchong and Longling, we collect temperature data from the website of Moderate Resolution Imaging Spectroradiometer (MODIS: https://modis.gsfc.nasa.gov/), which are available on an 8-day basis at 1 km spatial resolution. The average temperature over the area is used to estimate VCAP. Meanwhile, we collect the rainfall data from the website of Tropical Rainfall Measuring Mission (TRMM: https://pmm.nasa.gov/trmm), which are available on a three-hour basis at 0.25 deg spatial resolution. The cumulative rainfall is used to estimate VCAP. Finally, in the nonlinear stochastic model, both *x*_*t*_ and *y*_*t*_ are calculated as proportions out of the population size in Tengchong and Longling, respectively. Based on the sixth national census of China in 2010, there are 644 765 people in Tengchong and 277 319 people in Longling.

Some model parameters are set in advance based on existing studies as shown in Table 1, while other parameters *θ*={*β*,*r*,*α*_1_,*α*_2_,*ω*,*σ*} need to be inferred using the PMCMC algorithm. According to the Bayesian inference method, to reduce the influence of the priors on the posterior of *θ*, it is better to assume the uninformative prior distributions. Therefore, in our case studies, we assume the parameters in *θ* are independent of each other, and set the prior distribution of each parameter as an uninformative uniform distribution. Through careful testing, we set *β*∼*U*(0,1), *r*∼*U*(0,1), *α*_1_∼*U*(0,9), *α*_2_∼*U*(0,15), 1/*ω*∼*U*(20,28), and *σ*∼*U*(8,20). The initial value of each parameter is randomly generated based on its prior distribution. Moreover, with careful pre-testing, the proposal distribution of each parameter is set as follows: *q*(*β*^∗^|*β*)=*norm*(*β*^∗^|*β*,0.0022), $q(\alpha _{1}^{*}|\alpha _{1})=norm(\alpha _{1}^{*}|\alpha _{1},0.3)$, $q(\alpha _{2}^{*}|\alpha _{2})=norm(\alpha _{2}^{*}|\alpha _{2},0.32)$, *q*(*r*^∗^|*r*)=*norm*(*r*^∗^|*r*,0.016), *q*((1/*ω*)^∗^|1/*ω*)=*norm*((1/*ω*)^∗^|1/*ω*,0.23), and *q*(*σ*^∗^|*σ*)=*norm*(*σ*^∗^|*σ*,0.018). Since the interval of each prior covers almost all possible values of corresponding parameter, such settings have little effect on the inference results as long as the number of iterations is enough. In our experiments, the PMCMC algorithm is run for 500 000 iterations, following a discarded burn-in of 50 000 iterations. Finally, the posterior of each parameter is built upon the last 90% iterations.

## Results

### Parameter inference

**Fig. 1:**
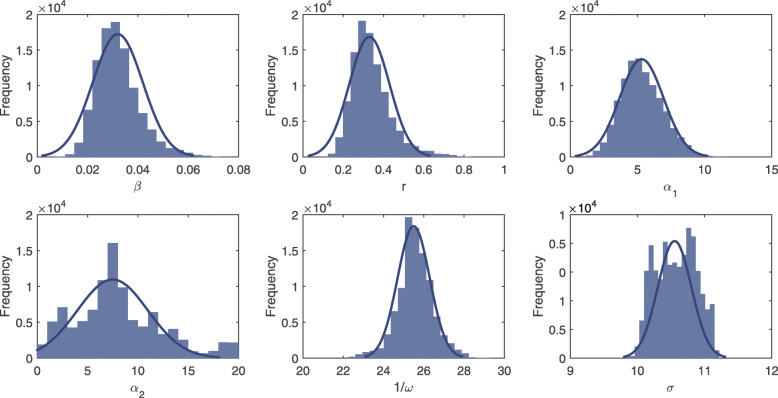
The estimated posterior density for all unknown parameters in the data-driven stochastic model using time series of malaria prevalence in Tengchong county. The horizontal axis represents the sampled values of each parameter based on the PMCMC algorithm, and the vertical axis represents the frequency at which the corresponding values appear in all samples

**Fig. 2:**
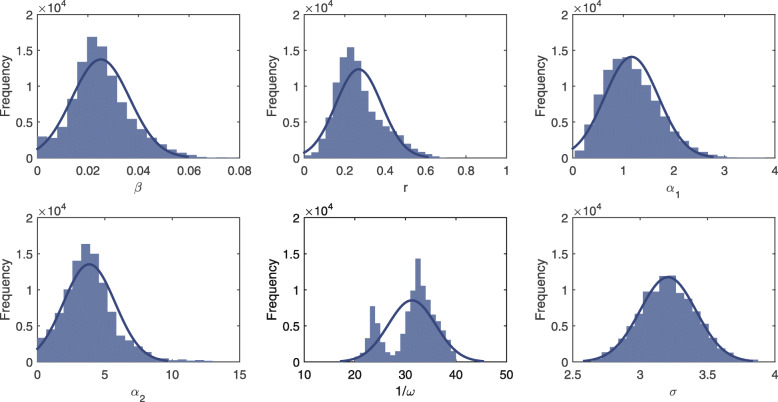
The estimated posterior density for all unknown parameters in the data-driven stochastic model using time series of malaria prevalence in Longling county. The horizontal axis represents the sampled values of each parameter based on the PMCMC algorithm, and the vertical axis represents the frequency at which the corresponding values appear in all samples

The set of model parameters *θ*={*β*,*r*,*α*_1_,*α*_2_,*ω*,*σ*} are inferred by sampling the values of each parameter that approximate the posterior distribution *p*(*θ*,**x**_1:*τ*_|**y**_1:*τ*_). Initially, the prior of each parameter is set to be a uniform distribution. During the updating process of the PMCMC algorithm, a set of parameter values will be sampled through the Monte Carlo simulation. Figures 1 and 2 show estimated posterior density for all unknown parameters in the data-driven stochastic model based on time series of malaria prevalence in Tengchong and Longling, respectively. The more the density conforms to the normal distribution with small variance, the more stable the parameter is estimated. It can be observed that the histograms of both mutual transmission efficiency *β* and human recovery rate *r* are subject to the normal distribution with small variance. Moreover, the estimates of *β* and *r* in both counties are very similar. The results indicate that our model can provide better estimations for transmission-related parameters. Besides, the parameter *α*_1_ can also be well estimated in both counties, indicating a strong correlation between the real-world observations and the data-driven malaria transmission model. In other words, it is reasonable to model the dynamics of malaria transmission based on the notions of VCAP and EIR by involving dynamically changing meteorological factors.

**Fig. 3:**
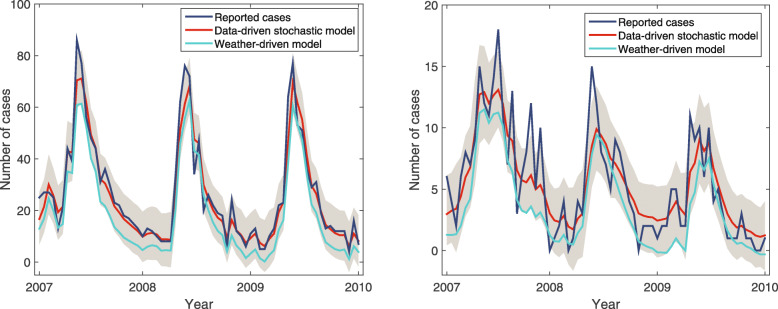
An illustration of the fitting results of the proposed data-driven stochastic model and the weather-driven model. The fitting results of Tengchong are shown on the left and Longling on the right. The blue line shows the real number of *P. vivax* cases from January 1, 2007 to December 31, 2009. The gray shadow covers the 95% percentiles of output samples *y*_1:*τ*_ based on the data-driven stochastic model, and the red line represents their average value. The light green line represents the estimation of *α*_1_**x**_1:*τ*_ based on the weather-driven model

With respect to the seasonality-related parameter, 1/*ω* reflects the cycle of peak values in the model. It is expected that the value of 1/*ω* should be around 23 because each calendar year is separated into 23 time windows in our case study. For the Tengchong county, the result in Fig. 1 shows that the estimate of 1/*ω* approximates to a normal distribution, indicating a stable estimation. However, the mean value is slightly larger than 23. Such an estimate is acceptable because the number of malaria cases is relatively small during several time windows across two consecutive calendar years. Moreover, since only three years of prevalence data are used for training the proposed model, no precise seasonal period can be found from real data. For the Longling county, the result in Fig. 2 shows that the estimate of 1/*ω* oscillates between the two values. The reason is that there are multiple peaks in the time series of Longling in a year (the blue line in Fig. 3), which may affect the estimation of 1/*ω*. However, these peaks cannot reflect the real seasonality of *P. vivax* infections in Longling because the number of *P. vivax* cases in Longling is much smaller than that in Tengchong. The increase or decrease of a small number of cases will cause severe fluctuations in the time series, which may disrupt the seasonality of the time series.

The estimates of *σ* in both counties are relatively large (see Figs. 1 and 2). The reason is that in the stochastic model, we assume that the number of imported malaria cases is subject to a strict periodic function. While in reality, imported cases are caused by human movement and many other factors, which are too complicated to be predicted. For the Tengchong county, the estimates of parameter *α*_2_ fluctuate in the region [0,20], and the samples of *σ* are relatively large and vary in the region [10,12]. While for the Longling county, the estimate of *α*_2_ is relatively stable and the samples of *σ* vary in the region [2.5,4]. The reason is that the number of *P. vivax* in Tengchong is much larger than that in Longling, and the seasonality of malaria cases in Tengchong is more obvious. The results suggest that in order to build more accurate models, one of the most important issues is to investigate the impact factors that are related to the number of imported cases.

### Fitting results

The fitting results of the data-driven stochastic model and the weather-driven model are shown in Fig. 3. Specifically, the data-driven model is trained using the PMCMC algorithm based on time series of *P. vivax* prevalence data in Tengchong and Longling from January 1, 2007, to December 31, 2009 (i.e., the blue line in Fig. 3). According to the PMCMC algorithm, a sampled sequence of the hidden state **x**_1:*τ*_ will be generated at each iteration. Following a discarded burn-in of 50 000 iterations, we sample **x**_1:*τ*_ every 20 iterations from the last 90% iterations, and calculate corresponding **y**_1:*τ*_ with the sampled model parameters. The gray shadow shows the region of 95% percentiles of output samples **y**_1:*τ*_, and the red line represents the average value of the output samples. It can be observed that the proposed stochastic model can well fit the real-world malaria prevalence data in both counties. Moreover, to evaluate the effect of meteorological factors on the *P. vivax* transmission, we also plot the estimation results of the weather-driven transmission model *α*_1_**x**_1:*τ*_ (the light green line in Fig. 3). It can be observed that the weather-driven model can reflect the trend of *P. vivax* infections in both counties, which verifies the important role of temperature and rainfall in malaria transmission dynamics.

**Fig. 4:**
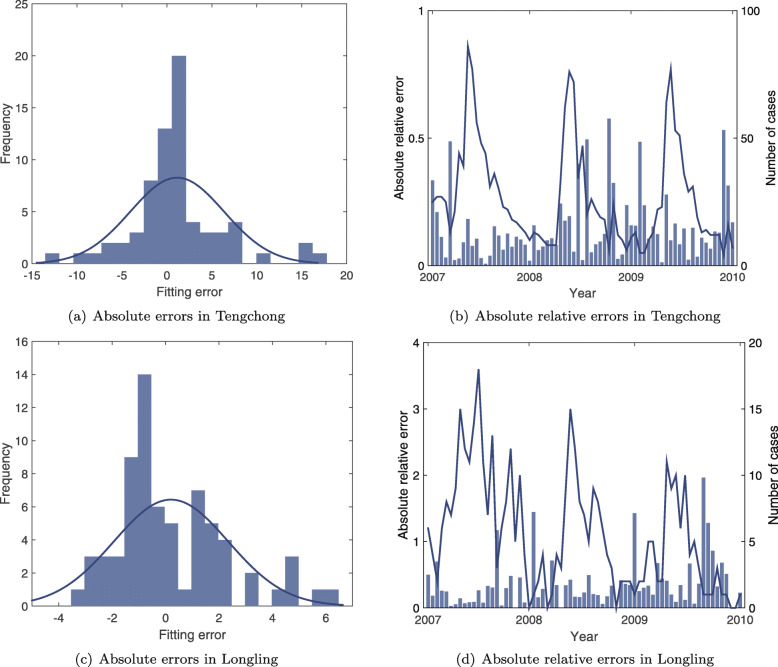
An illustration of the fitting errors of the data-driven stochastic model. The model is trained using the PMCMC algorithm based on time series of *P. vivax* prevalence data in Tengchong and Longling from January 1, 2007, to December 31, 2009. **a** The histogram of absolute fitting errors in Tengchong; **b** The absolute values of relative fitting errors in Tengchong over the three years; **c** The histogram of absolute fitting errors in Longling; and (**d**) The absolute values of relative fitting errors in Longling over the three years

We further evaluate the performance of the proposed models by examining the fitting results in Tengchong and Longling over the three years. Table 2 shows the comparison between the weather-driven model and the data-driven model in terms of the root-mean-square error (RMSE), the mean squared error (MSE), the mean absolute error (MAE), and the R-squared value. The results indicate that by including a periodic function to reflect the seasonality of malaria cases, the data-driven model can achieve better performance than the weather-driven model. Moreover, the data-driven model can better interpret the time series of *P. vivax* cases in Tengchong with *R*^2^=0.9323. However, even though the values of RMSE, MSE, and MAE of Longling are much smaller than that of Tengchong, the *R*^2^ value of Longling is smaller with *R*^2^=0.7609. The reason is that the number of reported cases in Longling is much less than that in Tengchong. A small change in the number of reported cases will result in large fluctuations in the time series of Longling county. Therefore, it is relatively difficult to make an accurate prediction because the periodicity is easily disturbed (see Fig. 2). Figure 4a and c illustrate the histogram of absolute fitting errors of the data-driven stochastic model in both counties over the three years. It can be observed that most fitting errors are very small, and the error histogram is subject to normal distributions with a mean of zero. The results indicate that the proposed data-driven stochastic model is well trained by the PMCMC algorithm. Because the number of cases varies dramatically, we further analyze the relative fitting errors of the data-driven model from January 1, 2007, to December 31, 2009. As shown in Fig. 4b and d, the blue bars represent the absolute values of relative fitting errors, and the blue line is the real number of *P. vivax* cases over time. An interesting observation is that the relative errors of our model are always small unless the real number of *P. vivax* cases changes unexpectedly. In other words, it is those sudden changes in time series of malaria cases that result in the fitting errors of the proposed model.

**Table 2:** The fitting results of the proposed models in Tengchong and Longling

County	Model	RMSE	MSE	MAE	R-squared
Tengchong	Weather-driven model	9.2986	86.4642	7.9169	0.8129
	Data-driven model	5.2562	27.6277	3.5287	0.9323
Longling	Weather-driven model	3.0427	9.2577	2.3347	0.6003
	Data-driven model	2.1368	4.5659	1.6566	0.7609

^1^RMSE: root-mean-square error; MSE: mean squared error; MAE: mean absolute error

### Prediction results

**Fig. 5:**
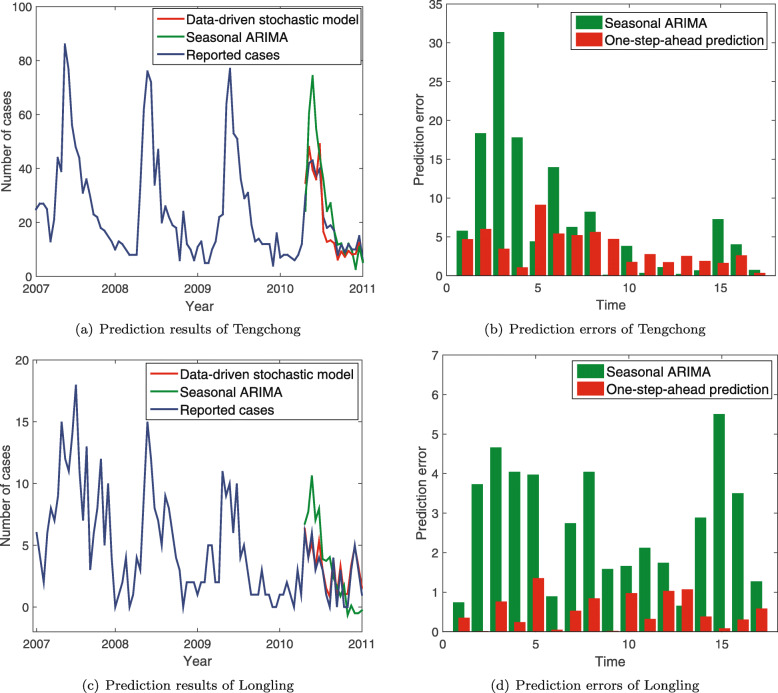
The comparison of prediction results between the one-step-ahead prediction model and the SARIMA model. **a** The predicted number of *P. vivax* infections in Tengchong; **b** The absolute prediction errors of Tengchong; **c** The predicted *P. vivax* infections of Longling; and **d** The absolute prediction errors of Longling. The red line presents the results of the one-step-ahead prediction model; the green line represents the results of the SARIMA model; the blue line is the real number of *P. vivax* cases

Because time series of malaria prevalence data in both counties have periodic characteristics, we evaluate the forecasting capability of the one-step-ahead prediction model by comparing it with the seasonal autoregressive integrated moving average (SARIMA) model. A seasonal ARIMA model is formed by including additional seasonal terms in the ARIMA, which involve backshifts of the seasonal period. According to the SARIMA model, the goal is to find optimal parameters of the *SARIMA*(*p*,*d*,*q*)(*P*,*D*,*Q*,*S*) model. Here, *p* is the trend autoregressive order, *d* is the trend difference order, and *q* is the trend moving average order. Meanwhile, *P*, *D*, and *Q* are seasonal autoregressive order, seasonal difference order, and seasonal moving average order, respectively. In our case study, *S*=23 is the number of time windows for a single seasonal period. Therefore, for the SARIMA model, there are a large number of combinations of parameters (*p*,*d*,*q*) and (*P*,*D*,*Q*). In our experiments, we assume that the value of each parameter can be taken from {0,1}. Using the *SARIMAX* function in the *statsmodels* package of Python, we try out all combinations of these parameters and choose the best fitting model based on the Akaike information criterion (AIC). In doing so, the model *SARIMA*(1,1,1)(1,1,0,23) are selected with the smallest AIC, which is further used to make predictions on the number of *P. vivax* infections in 2010. Figure 5 shows the comparison of prediction results between the one-step-ahead prediction model and the SARIMA model in Tengchong and Longling, respectively. It can be observed that comparing with the one-step-ahead model, the peak values predicted by the SARIMA model (the green curves in Fig. 5a and c) is much larger than the real number of *P. vivax* cases. The reason is that the SARIMA model makes statistical predictions based on the trend and seasonality of historical periodic events, which do not take into account the impact of risk factors. On the contrary, by considering dynamically changing meteorological factors in the data-driven stochastic model, the one-step-ahead prediction model can achieve better prediction results in both counties even though the number of *P.vivax* cases is relatively small in 2010 (the red curves in Fig. 5a and c). Accordingly, the prediction errors of the one-step-ahead prediction model are much smaller than that of the well-chosen SARIMA model (see Fig. 5b and d).

## Discussion

The natural malaria transmission depends on the complex interactions among three epidemiological entities: the parasite, the host, and the transmission agent (i.e., mosquitos). The transmission dynamics can also be affected by various factors, such as biological, human behavioral, demographic, socioeconomic, environmental and ecological factors, ranging from a microscopic scale to a macroscopic scale. For example, biological factors (e.g., acquisition of immunity) at the microscopic scale may determine the vulnerability of an individual to infection [46], while meteorological factors (e.g., temperature and rainfall) at the macroscopic scale are instrumental to the gonotrophic cycle length of mosquitoes [40]. More importantly, most impact factors change over time. Therefore, to quantitatively analyze the dynamics of malaria transmission, it would be necessary to involve various dynamically changing impact factors into the model. The proposed data-driven stochastic model adopts the notion of VCAP and EIR to characterize the impact of meteorological and demographic factors on the risk of malaria transmission, which makes it possible to quantitatively assess and predict the patterns of malaria transmission.

In reality, the exact values of many transmission-related parameters are very difficult to obtain through field study. For example, to measure the human blood index, the front-line staffs are required to regularly work as baits to count the number of bites by mosquitoes [37]. Even though, there are many other transmission-related parameters that are impossible to directly obtain, such as the mutual transmission efficiency *β* and the human recovery rate *r*. By training the data-driven stochastic model based on time series of malaria prevalence data, the transmission-related parameters *β* and *r* can be inferred approximately. The case studies in Tengchong and Longling have shown that the estimated value of mutual transmission efficiency *β* is in the region [0.015,0.040] and the human recovery rate *r* falls into the region [0.20,0.30]. Such estimates could help build more accurate malaria transmission models, and further, evaluate the force of infection and/or the basic reproduction number.

In the past, many statistical methods have been proposed to forecast the potential disease infections, such as the linear regression model, generalized additive model, and SARIMA. However, such methods focus purely on modeling the linear or nonlinear relationships between various impact factors and time series of historical disease prevalence data. They do not aim to unveil the underlying dynamics of disease transmission, and hence are limited in making an accurate prediction about potential infections when the transmission situations change dramatically. For example, in Fig. 5, the prediction results in Tengchong and Longling have shown that the SARIMA model inclines to overestimate the number of malaria infections in March and April due to the seasonality in the model. On the contrary, the proposed data-driven stochastic model can make a better prediction by modeling the dynamics of malaria transmission with dynamically changing impact factors and malaria prevalence data.

Although the proposed models can reveal certain dynamical patterns of malaria transmission, there are still several limitations in this work. First, although the values of two transmission-related parameters can be inferred based on the time series of historical prevalence data, many other parameters are still surveyed from literature. In the future, specific investigations are expected to be conducted in Tengchong and Longling, to reveal the relationships between environmental/meteorological factors and other transmission-related parameters (e.g., mosquito density per person). Second, in this paper, we simply assume the seasonality of the number of imported cases, which results in the large value of *σ* in Figs. 1 and 2. To further improve the prediction accuracy of the proposed model, it is expected to investigate the causality of imported cases at different locations, especially, the patterns of human movement [12, 24, 25]. Third, the VCAP in this paper is derived from the Macdonald model, where many processes, such as the relapses episodes of *P. vivax* and the time lag introduced by parasite incubation, are not considered. To develop a more comprehensive model, it would be helpful to take into consideration such critical dynamic processes. Fourth, the predictive ability of our model will decrease when the number of malaria cases is small. As shown in Fig. 3 and Table 2, the ability of our model to predict malaria infections in Longling is worse than that of Tengchong. In the future, more attention should be paid on assessing malaria transmission risks during the phase of pre-elimination. Finally, as more and more prevalence data are available, comparative studies can be conducted to reveal the similarities and differences in malaria transmission at different geographic locations.

## Conclusion

In this study, we have proposed a data-driven nonlinear stochastic model to (i) investigate the underlying malaria transmission dynamics, and (ii) predict the number of potential infections. Specifically, we have taken into account the impact of both meteorological factors on disease transmission and seasonal patterns of imported cases, where the notions of VCAP and EIR is used to relate the risk of malaria transmission to the dynamically changing temperature and rainfall. Concerning unknown parameters in the model, we have presented a particle MCMC algorithm to estimate model parameters based on time series of malaria prevalence. By applying our model to *P. vivax* transmission in Tengchong and Longling, Yunnan province, China, we have demonstrated its ability to make a reasonable estimation for model parameters, which help better understand the transmission dynamics of *P. vivax*. Further, based on the well-trained model, we have evaluated the forecasting capability of our one-step-ahead prediction method by making a comparison with the ASRIMA model in both counties.

## Data Availability

Please contact author for data requests.

## Abbreviations

ARIMA: Autoregressive integrated moving average
PMCMC: Particle Markov chain Monte Carlo
VCAP: Vectorial capacity
EIR: Entomological inoculation rate
SMC: Sequential Monte Carlo
CISDCP: China information system for disease control and prevention
MODIS: Moderate resolution imaging spectroradiometer
TRMM: Tropical rainfall measuring mission
AIC: Akaike information criterion
